# Acute Aflatoxicosis Resulting in Fulminant Hepatic Failure and Rhabdomyolysis

**DOI:** 10.4021/gr2009.01.1254

**Published:** 2009-01-20

**Authors:** Nir Samuel, Yosef Ezri, Raymond Farah, Vacksman Igor, Amer Hussein, Orit Rubinshtein, Nimer Assy

**Affiliations:** a Department of Internal Medicine B, Ziv Medical Center, Safed, Israel; b Intensive Care Unit, Ziv Medical Center, Safed, Israel; c Department of Emergency Medicine, Rebekka Ziv Medical Center, Safed, Israel; d Liver Unit, Ziv Medical Centre, Safed, Israel; e Department of General Surgery, Ziv Medical Center, Safed, Israel; f Technion Institute, Faculty of medicine, Haifa, Israel

**Keywords:** aflatoxin, aflatoxicosis, Aspergillus, fulminant hepatic failure, rhabdomyolysis

## Abstract

**Background:**

Aflatoxins are known contaminants of foods. High dose exposure, particularly to Aflatoxin B_1_ (AFB_1_) may cause acute aflatoxicosis. Outbreaks have been reported in developing nations but are virtually un-documented in the developed world.

**Case report:**

A 28 year old, healthy male presented with nausea, vomiting and abdominal pain. The patient deteriorated rapidly to a state of agitation and shock. The clinical picture, encephalopathy and laboratory results indicated fulminant hepatic failure, rhabdomyolysis and multi-system organ failure. Canned food the patient consumed almost exclusively contained AFB_1_ at a level of 19.6 ppb. Alternate diagnoses were ruled out and a presumptive diagnosis of acute aflatoxicosis was made. After 45 days of intensive supportive therapy, the patient was discharged with no significant sequels.

**Conclusions:**

The diagnosis of aflatoxicosis was based on the clinical picture, the finding of high levels of AFB_1_ in foods the patient consumed, and after alternate diagnoses' were sufficiently excluded. We conclude that chronic exposure to moderately elevated levels of aflatoxin B_1_ may result in acute aflatoxicosis and fulminant hepatic failure.

## Introduction

Aflatoxins are a group of mycotoxins produced by the fungi *Aspergillus flavus* and *Aspergillus parasiticus* and are known contaminants of a variety of foods. Chronic, low dose exposure to aflatoxins is a well documented cause of liver malignancy and immunologic disturbances. Short term, high dose exposure, particularly to Aflatoxin B_1_ (AFB_1_), the most potent of these toxins, may cause acute aflatoxicosis. Aflatoxin poisoning may result in hemorrhagic liver necrosis, steatosis, bile duct proliferation and subsequent organ failure, with a mortality rate of close to 25% and may be higher [1, 2]. Aflatoxin exposure has also been suggested to contribute to the incidence of Reye and Reye-like syndromes [3]. Outbreaks of aflatoxicosis have been reported in developing nations around the world [2, 4, 5]. However, due to food manufacturing and monitoring standards, it is virtually un-documented in the developed world. We present here a case of suspected severe aflatoxicosis in a migrant Thai worker who consumed large quantities of contaminated food shipped to him from his native country.

## Case report

A 28 year old, healthy Thai male, employed in Israel as an agricultural worker, presented to the emergency room with complaints of nausea, vomiting and diffuse abdominal pain starting 3 days earlier. Past medical history was unremarkable and without mention of recent illness. On presentation, the patient was well oriented in no severe distress, physical examination was notable for epigastric tenderness with guarding. Heart rate was 116 beats/minute, blood pressure 94/59 mm/Hg and a temperature of 37.4°C. The patient was admitted to the surgical department for evaluation. A chest and abdomen CT revealed marked liver enlargement with fatty change, and minimal amounts of pleural and pericardial fluid. No surgical etiology was recognized and due to laboratory findings of renal failure (Creatinine 3.88 mg/dl BUN 119.7 mg/dl), the patient was transferred to the medical department.

Shortly after arrival, the patient's condition deteriorated rapidly to a state of agitation and shock and he was transferred to the intensive care unit. On arrival blood pressure measured 79/45 mm/Hg, pulse 170 beats/minute with episodes of SVT. In addition, the patient developed dyspnea, with a respiratory rate of 48 breaths/minute and anuria. Core temperature measured 33.1°C. Examination revealed marked peripheral cyanosis, distended jugular veins with cool extremities. Air entry to both lungs was reduced and palpation of the abdomen was noted for tenderness and an enlarged liver. Laboratory analysis showed lactic acidosis (pH 7.22, lactate 129 mg/dl), renal failure (creatinine 2.11 mg/dl), and rhabdomyolysis (CPK 3,205 IU/l). Liver function tests and coagulation indices were also disturbed ( total bilirubin 2.74, direct bilirubin 1.03 mg/dl, AST 1169 IU/l, ALT 890 IU/l, LDH 1743 IU/l, INR 1.7, factor V level 18%,). Erythrocyte sedimentation rate was within normal limits. Factor V levels continued to decline to a nadir of 5%. The above laboratory results, in combination with encephalopathy, indicated fulminant hepatic failure accompanied by multi-system organ failure.

The patient failed a trial of positive pressure ventilation and was subsequently intubated and ventilated mechanically. A Swan-Ganz catheter was inserted and fluid resuscitation started, steroids and vasopressors were given. Echocardiography was used to rule out pericardial tamponade; normal ventricular and valvular function was demonstrated. Continuous hemodiafiltration was started and switched to standard hemodialysis a week later. A tracheotomy was later performed due to the need for prolonged ventilation. Blood, urine, sputum and peritoneal fluid aspirate were cultured and gram and Ziell-Nielsen stained. Empiric antibiotic therapy was then started. Culture results were negative. Serologic testing for acute or chronic infection with viral hepatitis types A, B and C, HIV, Epstein-Barr virus, Cytomegalovirus returned negative. Further serologic testing for *Ricketsia conorii*, *Coxiella burnetii* were also negative. A thick film smear for blood-borne parasites and stool collection for ova and parasites were negative. Due to a high osmolar gap, levels of methanol, ethylene glycol and acetyl salicylic acid were checked and no traces were found.

A health bureau inspector was sent to the residence of the patient and found a stock of canned food the patient consumed almost exclusively for a period of months. Specimens were examined at the ministry of health. AFB_1_ was found at a level of 19.6 ppb (allowed level < 5 ppb). The patient's condition gradually improved on supportive treatment. Vasopressors were stopped and dialysis discontinued after blood pressure, renal function and CPK values returned to normal. Liver function tests improved markedly. After 21 days of mechanical ventilation the patient was weaned and the tracheotomy closed on the 36th day. The patient was returned to the medical department for observation and physiotherapy. No apparent sequels were observed other than mild residual hypertension. The patient was discharged on day 45.

## Discussion

Disease due to aflatoxin exposure was first recognized in livestock a few decades ago with similar patterns of disease apparent in different species. Susceptibility to aflatoxin has been noted to vary considerably between species and between different individuals, including man. Of note is the higher susceptibility in the young. The level of aflatoxin exposure needed to cause acute aflatoxicosis has not, thus far, been determined in humans. Aflatoxin is recognized by the FDA as an unavoidable contaminant of food and standards have been set for its level in commercially distributed products. These standards are not applicable in the developing world where food manufacturing technology and surveillance is lacking. As a result, billions of people are exposed to uncontrolled amounts of toxin [1]. A literature search reflects this dichotomy and reports of aflatoxicosis emerge mostly during sporadic epidemics in developing countries [2, 4, 5], perhaps due to the fact that individual cases are under-diagnosed.

In this case, the presumptive diagnosis of aflatoxicosis was based on the clinical picture, the finding of high levels of AFB_1_ in foods the patient consumed almost exclusively, and after alternate diagnoses' were sufficiently excluded.

The combination of fulminant hepatic failure and rhabdomyolysis should raise the suspicion of a toxic etiology. The marked discrepancy between extremely low factor V levels and only moderately disturbed liver function tests and coagulation indices may be another clue to this etiology. The full recovery without sequels from a state of multi-system organ failure after receiving intensive, yet basically supportive therapy, is again compatible with poisoning. Although the elevated levels of AFB_1 _in the specimens collected, were not as high as in previously reported cases [2, 4], we believe that, given the known variable susceptibility to AFB_1_ and the fact that the level of AFB_1_ in the specimen collected may be an underestimate, it may well be the cause of disease.

We conclude that chronic exposure to moderately elevated levels of aflatoxin B_1_ may result in acute aflatoxicosis and fulminant hepatic failure. This case also illustrates the appearance of disease in a part of the world where it is practically unknown, due to immigration of people and the shipment of goods between the developed and developing world. This epidemiologic pattern will most likely become increasingly more prevalent and will require clinicians to consider a wider range of diagnoses in a given clinical presentation.

## Abbreviations

AFB_1_: aflatoxin B_1_
CT: computerized tomography
SVT: supra-ventricular tachycardia
CPK: creatinine phosphokinase
FDA: Food and Drug Administration (US)
